# 1H NMR Fingerprinting of Soybean Extracts, with Emphasis on Identification and Quantification of Isoflavones

**DOI:** 10.3390/nu2030280

**Published:** 2010-02-26

**Authors:** Augusta Caligiani, Gerardo Palla, Annalisa Maietti, Martina Cirlini, Vincenzo Brandolini

**Affiliations:** 1 Dipartimento di Chimica Organica e Industriale, Università di Parma, Via Usberti 17A, 43100-Parma, Italy; Email: gerardo.palla@unipr.it (G.P.); martina.cirlini@nemo.unipr.it (M.C.); 2 Dipartimento di Scienze Farmaceutiche, Università di Ferrara, Via Fossato di Mortara 17/19, 44100 Ferrara, Italy; Email: mttnls@unife.it (A.M.); bnv@unife.it (V.B.)

**Keywords:** 1H NMR, soybean, isoflavones, fingerprinting

## Abstract

1H NMR spectra were recorded of methanolic extracts of seven soybean varieties (*Glycine max.*), cultivated using traditional and organic farming techniques. It was possible to identify signals belonging to the groups of amino acids, carbohydrates, organic acids and aromatic substances in the spectra. In the aromatic zone, the isoflavone signals were of particular interest: genistein, daidzein, genistin, daidzin, malonylgenistin, acetylgenistin, malonyldaidzin signals were assigned and these compounds were quantified, resulting in accordance with published data, and further demonstrating the potential of the NMR technique in food science.

## 1. Introduction

**Figure 1:**
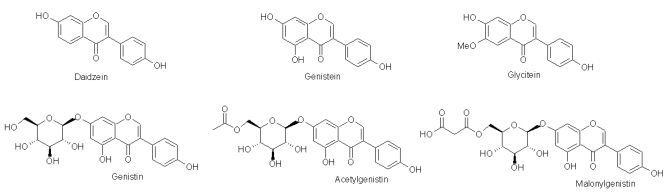
Soybean isoflavone aglycones and genistein conjugated forms.

Soybean (*Glycine max L*.), traditionally produced and consumed in China, is today one of the most important agricultural commodities in the world, cultivated mainly for its high content of protein and oil. In recent decades, several studies have shown the health benefits of soy components; regular consumption of soy foods can reduce the incidence of breast, colon, and prostate cancers [1], prevent heart disease and osteoporosis, and reduce menopausal symptoms [2]. These discoveries have resulted in the development and commercialization of many functional foods and food supplements based on soy ingredients. Isoflavones are most likely the components responsible for the health benefits of  soy [3]. Isoflavones belong to a group of compounds that share a basic structure consisting of two benzyl rings joined by a three-carbon bridge. Isoflavones in soybeans and soy products exist as aglycones (daidzein, genistein, and glycitein), 7-O-β-glucosides and two glucoside conjugate forms, acetylglucosides and malonylglucosides (Figure 1). The 7-O-β-glucosides are commonly known as daidzin, genistin or glycitin. Soybeans contain high amounts of isoflavones, normally in the range of 1–4 mg/g dry weight [4]. 

Soybean components, in particular isoflavones, can widely vary, depending on the varieties, cultivation technique *etc.* [5], so it is important to develop specific and rapid analytical methods for the characterization of soybeans and soy products. 

NMR spectroscopy has become increasingly important in food science [6], both as a fingerprinting technique [7] and as quantitative analysis tool [8,9]. This advance in the development of NMR methods in food characterization and control is mainly due to the simple preparation of samples, the speed of analysis and the possibility of obtaining structural information in a complex food matrix.

In this experimental work, the 1H NMR spectra of methanolic extracts of different defatted soybean varieties, coming from traditional and organic farming, were recorded, in order to determine the potential of the NMR technique for soybean composition control.

## 2. Results and Discussion

### 2.1. 1H NMR Signal Assignment

**Figure 2:**
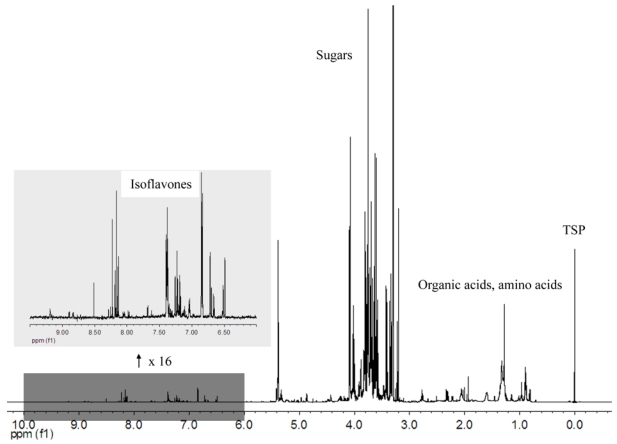
1H NMR spectrum of a soybean methanolic extract, with insert (x16) showing the aromatic region, mainly constituted of isoflavones signals

Figure 2 shows the 1H NMR spectrum of the methanolic extract of a soybean defatted meal, registered with PRESAT water suppression. Many signals of the spectra (alanine, organic acids, choline, sugars and isoflavones) were identified and are reported in  1.  A  group  of  signals  were  tentatively  attributed  to  soy  saponins  characterized  by  the  linkage  to  a  2,3-dihydro-2,5-dihydroxy-6-methyl-4H-pyran-4-one (DDMP) moiety.

The identification of the substances was made by recording NMR spectra of pure compounds, or by comparison with spectra previously reported in literature or in databases. For isoflavones, a further confirmation of the signal assignments was obtained by spiking the soybean extracts with appropriate standards, as reported in section 2.2. The identification of DDMP saponins signals was based on the 1H NMR data previously reported for soy DDMP saponins recorded in deuterated DMSO (10).

Table 1 reports only the observable and not overlapping signals, other signals were present in the zone 0–2.4 ppm, but strongly overlapped with signals of lipids.

**Table 1:** Characteristics of 1H NMR signals observable in the zone 1-9 ppm of soybean methanolic extract.

δ (ppm)	Multiplicity	Compound	Group	J (Hz)
***Lipids***				
0.713	s	Beta-sitosterol	CH_3_	-
0.732	s	Stigmasterol	CH_3_	-
0.889-0.923	triplets	Fatty acids	CH_3_	
1.281-1.374		Fatty acids	CH_2_	
1.607		Fatty acids	CβH_2_	
2.082		Fatty acids	CH_3_ (allylic)	
2.321		Fatty acids	CαH_2_	
***Organic acids, amino acids and alcohols***				
1.467	d	Alanine	CβH_3_	7.22
1.952	s	Acetic acid	C2H_3_	-
2.662	s	Succinic acid	C2H_2_+C3H_2_	-
2.950	dd	Malic acid	C3H_a_	16.31; 4.27
3.231	s	Malonic acid	C2H_2_	-
3.220	s	Choline	N(CH_3_)_3_	-
***Saponines***				
1.991	s	DDMP Saponin?	C6’CH_3_	-
2.022	s	DDMP Saponin?	C6’CH_3_	-
2.041	s	DDMP Saponin?	C6’CH_3_	-
2.069	s	DDMP Saponin?	C6’CH_3_	-
***Sugars***				
3.346	t	Sucrose		9.43
3.420	dd	Sucrose		3.80, 9.73
3.453	dd	Stachiose		3.71, 9.78
3.748	m	Sucrose		-
4.021	t	Sucrose		7.23
4.076	s	Sucrose		-
4.090	s	Sucrose		-
4.869	dd	Stachiose		3.58, 13.46
4.965	dd	Raffinose		3.48, 6.86
5.390	d	Sucrose		3.77
5.424	d	Stachiose		3.88
Isoflavones*				
6.930	dd	Daidzein	C6H	8.83; 2.19
7.242	d	Daidzin	C8H	2.23
7.251	d	Malonyldaidzin	C8H	2.27
6.221	d	Genistein	C6H	2.17
6.518	d	Genistin	C6H	2.19
6.495	d	Malonylgenistin	C6H	2.18
6.529	d	Acetylgenistin	C6H	2.19

*Signals chosen for quantification of isoflavones in soybean extracts

### 2.2. Assignment of Isoflavones Signals

**Figure 3:**
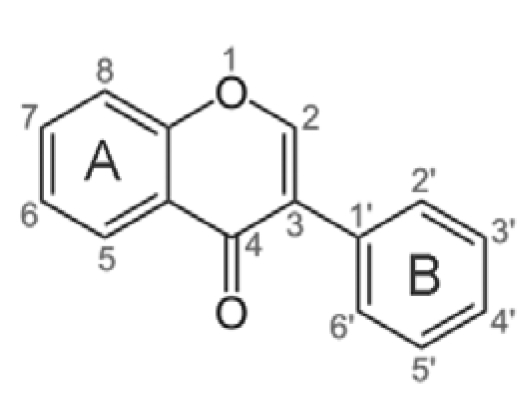
Numeration of atoms in the isoflavone skeleton.

One of the most interesting classes of substances in soybean is isoflavones. These compounds are characterized by the polycyclic structure reported in Figure 3, with A and B aromatic rings that give signals in a region of the 1H NMR spectrum (up to 6 ppm) generally free from interferences. 

The spectra of soybean samples were carefully studied in this zone (6–10 ppm) in order to establish that the isoflavones signals did not overlap and were reliable for quantitative analysis.

In order to obtain reliable signal assignment, the 1H NMR spectra of the standard solutions of genistein, daidzein, genistin and daidzin were recorded using the same conditions as the samples. The complete signal assignment of the standard compounds is reported in Table 2. Atoms were designated according to the numbering scheme of the carbon skeleton defined in Figure 3.

Comparison of the chemical shifts of aglycones (daidzein and genistein) and of their 7-O-glucosides (daidzin and genistin), shows that hydrogens of B ring are not influenced by the bond with glucose, while those of ring A are shifted to lower fields: for daidzin shifts were 0.28 ppm for hydrogen linked to C6, 0.38 ppm for hydrogen linked to C8, 0.10 ppm for hydrogen linked to C5, 0.07 ppm for hydrogen linked to C2. For genistin they were 0.29 ppm for hydrogen linked to C6, 0.36 ppm for hydrogen linked to C8 and 0.07 ppm for hydrogen linked to C2.

The isoflavones signals were then identified in the 1H NMR spectra of the soybean extracts. To obtain unambiguous assignments, samples were spiked with standards. As previously reported, the aromatic zone in the 1H NMR spectrum was rather free from interferences and the isoflavone signals were easily detectable. A specific NMR signal was selected for each isoflavone, and its area was utilized for the quantitative analysis. The signals chosen are those reported in the last part of Table 1.

**Figure 4:**
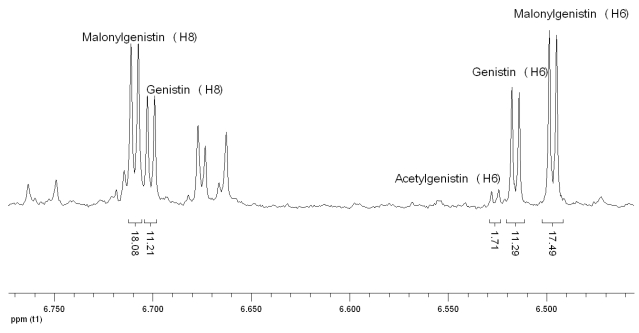
1H NMR signals of hydrogen atoms linked to C6 and C8 of genistin and its conjugated forms.

For the malonylglucosides and acetylglucosides forms of isoflavones not available as standards, the signal assignment was performed on the basis of the specific characteristics of H signals (chemical shifts, multiplicities and coupling constants), assuming little shifts of aromatic signals in respect to simple glycosides, due to the binding of acids to the C6-hydroxy group of glucose. Figure 4 shows the group of signals attributed to H6 of acetylgenistin and malonylgenistin, on the basis of the following consideration. It is common knowledge that, in general, the linkage of the acetyl group to a molecule causes shifts of 1H NMR signals to lower field, and this property was previously demonstrated also in the specific case of acetic esters of glucose (11). So it was reasonable to assume that the deshielding effect propagates also to the isoflavone moiety, and the signal at 6.529 ppm, showing the same multiplicity and coupling constant of H6 of genistin, was attributed to H6 of acetylgenistin. A similar shift was also previously reported for genistin and acetylgenistin 1H NMR spectra recorded in DMSO (12). A similar signal at 6.495 ppm was therefore assigned to malonylgenistin. In order to explain the unexpected shift to a higher field induced by the malonyl group to H6 we suppose that the free acid group of malonic acid could make weak interaction with the hydroxyl group at position 5 of genistin, *via* hydrogen bonding. The modified electron distribution could cause a little shielding near the H6, hence a shift to high fields. This hypothesis is supported by the fact that only H6 of malonylgenistin shows this behavior; the other observable hydrogen that can be attributed to malonylgenistin (H8, 6.71 ppm, Figure 4) is shifted to low fields with respect to the genistin signal as expected, like all the observable hydrogens of malonyl daidzin, which does not have the hydroxyl group in position 5. The signal at 7.250 ppm was chosen for the quantification of malonyldaidzin, while it was not possible to identify a clearly separate signal for acetyldaidzin. No forms of glycitein were detected in the spectra, probably because of their low abundance in the soybean varieties analyzed.

**Table 2:** Assignment of isoflavones standard compounds.

Compound	Group	δ (ppm)	Multiplicity	J (Hz)
Daidzein	C3’H, C5’H	6.841	d	8.54
	C8H	6.861	d	2.18
	C6H	6.930	dd	8.83; 2.19
	C2’H, C6’H	7.355	d	8.53
	C5H	8.042	d	8.78
	C2H	8.110	s	-
Daidzin	C3’H, C5’H	6.843	d	8.77
	C6H	7.212	dd	2.34; 8.89
	C8H	7.242	d	2.23
	C2’H, C6’H	7.370	d	8.77
	C5H	8.143	d	8.87
	C2H	8.187	s	-
	Glucose (anomeric)	5.090	d	7.56
	Glucose (other signals)	3.38–3.94	-	-
Genistein	C6H	6.221	d	2.17
	C8H	6.333	d	2.17
	C3’H, C5’H	6.840	d	8.72
	C2’H, C6’H	7.360	d	8.73
	C2H	8.050	s	-
Genistin	C6H	6.518	d	2.19
	C8H	6.692	d	2.06
	C3’H, C5’H	6.840	d	8.75
	C2’H, C6’H	7.387	d	8.76
	C2H	8.120	s	-
	Glucose (anomeric)	5.041	d	7.61
	Glucose (other signals)	3.38–3.94	-	-

### 2.3. 1H NMR Quantification of Isoflavones in Soybean Samples

For a reliable quantitative analysis by 1H NMR it is very important to improve the phase and the baseline; the phase was corrected manually for each sample, and a polynomial baseline correction was applied over the entire spectral range. In some cases a further manual adjustment of the baseline was performed. The manual integration of the selected signals and the comparison with TSP area enabled the quantitative determination of the soybean isoflavones. The data obtained are reported in Table 3.

The data allow some preliminary conclusions to be made: the most abundant isoflavones in the soybean varieties analyzed are malonylglucosides, followed by glucosides, which is in agreement with the data previously reported (4,5). Aglycone forms are present in very low concentration especially in samples from organic farming. Total isoflavones content of the soybean varieties analyzed included a large concentration range, and soybeans from traditional farming present, in general, a higher content of isoflavones, except for varieties T5 and T7.

The quantities of isoflavones obtained from soybean samples are in accordance with previously published data [4]. 

**Table 3:** Quantity (mg/kg of fresh soybean) of isoflavones in soybean varieties (1–7), determined by 1H NMR.

	Genistein	Genistin	Malonyl-genistin	Acetyl-genistin	Daidzein	Daidzin	Malonyl-daidzin	Total
**Soybean varieties from traditional farming**								
T1	20.6 ± 0.3	967 ± 32	531 ± 17	56 ± 4	56 ± 3	968 ±24	586 ± 8	3,184
T2	46.3 ± 0.5	489 ± 15	667 ± 20	74 ± 5	75 ± 1	341 ± 3	539 ± 10	2,231
T3	19.4 ± 0.3	680 ± 18	422 ± 13	55 ± 3	28.3 ± 0.5	431 ± 11	279 ± 5	1,914
T4	27.3 ± 0.4	578 ± 20	1045 ± 23	98 ± 7	25 ± 1	324 ± 8	547 ± 9	2,644
T5	26.3 ± 0.4	274 ± 14	324 ± 10	36 ± 2	42.6 ± 0.8	206 ± 5	317 ± 6	1,225
T6	24.2 ± 0.8	467 ± 16	727 ± 28	87 ± 7	35.1 ± 0.7	440 ± 10	682 ± 20	2,462
T7	6.5 ± 0.2	196 ± 10	464 ± 14	44 ± 3	18.7 ± 0.4	176 ± 5	456 ± 9	1,361
**Mean **	**24.4**	**521**	**597**	**64**	**40.1**	**412**	**486**	**2,146**
**Soybean varieties from organic farming**								
O1	0.34 ± 0.05	332 ± 12	234 ± 8	26 ± 2	0.33 ± 0.01	335 ± 9	234 ± 4	1,161
O2	0.35 ± 0.05	188 ± 8	571 ±17	57 ± 4	0.32 ± 0.02	249 ± 6	720 ± 20	1,785
O3	n.d	215 ± 8	191 ± 7	14 ± 1	n.d	294 ± 7	250 ± 6	964
O4	n.d	73 ± 4	189 ± 5	18 ± 1	n.d	90 ± 3	211 ± 3	581
O5	n.d	228 ± 9	555 ± 18	60 ± 3	n.d	187 ±5	479 ± 10	1,609
O6	n.d	157 ± 6	483 ± 14	49 ± 4	n.d	158 ± 6	505 ± 8	1,352
O7	n.d	384 ± 16	1117 ± 25	119 ± 10	0.32 ± 0.02	269 ± 8	872 ± 18	2,761
**Mean **	**0.34**	**225**	**477**	**49**	**0.32**	**226**	**481**	**1,459**

## 3. Experimental Section

***Samples***. 1H NMR analyses were performed on the seeds of seven experimental soy varieties (Table 4), each coming from two different cultivation techniques (traditional and organic).

***Materials***. Ethyl ether and methanol for extraction were purchased from Carlo Erba reagenti (Milan, Italy), standards of genistein, daidzein, genistin and daidzin, deuterated methanol (CD_3_OD) and 3-(trimethylsilyl)-propionate-d4 (TSP, internal standard for NMR analysis) were purchased from Sigma-Aldrich (Milan, Italy).

***Sample preparation***. Finely ground soybean samples were defatted by ethyl ether extraction in an automated Soxhlet apparatus (VELP, Milan). 0.5 g of the soybean defatted meal was then extracted for 1 hour at room temperature under magnetic stirring with 25 mL of a mixture methanol/water (4:1 v/v). The solution was filtered using filter paper and then a nylon filter (0.45 μm), taken to dryness and dissolved in 1 mL of CD3OD containing 0.02 mg of 3-(trimethylsilyl)-propionate-d4 (TSP) and transferred in a 5 mm NMR sample tube. TSP was used as both a chemical shift reference (δ = 0) and internal standard for the quantitative analysis. 

***1H NMR conditions***. Spectra were recorded on a VARIAN INOVA-600 MHz spectrometer, operating at 14.1 T and equipped with a 5 mm-triple resonance inverse probe. The 1H NMR spectra were acquired with low power selective water signal irradiation during relaxation delay (d1). Data were collected at 308 K, with sample rotation (20 Hz), 32K complex points, using a 90° pulse length. 128 scans were acquired with a spectral width of 8000 Hz, an acquisition time of 1.892 s and a recycle delay of 1.5 s. The NMR spectra were processed by MestreC software. The spectra were Fourier transformed with FT size of 64K and 0.3 Hz line-broadening factor, phased and baseline corrected, and referenced to the TSP peak (0 ppm). Phase correction was performed manually for each sample, and a polynomial baseline correction was applied over the entire spectral range. 

**Table 4:** Soybean varieties analyzed.

Samples from traditional farming	Samples from organic farming	Varieties
T1	O1	GORIZIANA
T2	O2	561/22A
T3	O3	LEONOR
T4	O4	REGIR
T5	O5	ATLANTIC
T6	O6	SAPPORO
T7	O7	FLY

## 4. Conclusions

This study represents a further confirmation that 1H NMR is a powerful tool for the analysis of food matrices, offering the advantages of simple sample preparation and the rapidity of data acquisition. In the particular case of soybean, the approach presented could have many interesting applications, for example in studying the different distribution of metabolites in GMO soy, or for the screening of food and food supplements based on soy components (e.g., isoflavones supplements) present on the market. The method could also be useful for a rapid quantification of the main soybean isoflavones, as it requires minimal sample pre-treatment.
